# Editorial: Patterning tissue microenvironment for optimizing wound healing and endogenous regeneration

**DOI:** 10.3389/fbioe.2023.1341574

**Published:** 2023-12-01

**Authors:** Nadya Lumelsky, Rosemarie Hunziker, Melissa D. Krebs

**Affiliations:** ^1^ National Institutes of Health (NIH), Bethesda, MD, United States; ^2^ Advanced Regenerative Manufacturing Institute, Manchester, NH, United States; ^3^ Colorado School of Mines, Golden, CO, United States

**Keywords:** stem cells, wound microenvironment, tissue regeneration, biomolecule delivery, real time imaging

This Research Topic called for article contributions addressing a broad array of optimization strategies to induce and sustain endogenous wound healing and tissue regeneration (Lumelsky, 2021). The timeliness of this Topic stems from a series of recent advances in many areas of basic biology and bioengineering. Among others, these advances include increased elucidation of the nature and properties of stem and progenitor cells; improved understanding of the composition and structure of stem cell niches; development of next-generation native and synthetic biomaterials with a capacity to control cellular responses as well as the properties and architecture of extracellular matrices (ECM); sophisticated biomolecule delivery that can temporally and spatially pattern the composition and structure of cellular microenvironment; progress in gene editing and genetic engineering; and high-resolution imaging and cell tracking.

In response to this call, we received seven articles that included four reviews and three original research papers emphasizing a spectrum of robust and diverse effort in this emerging and fast-developing field (Evano et al., 2023; Freedman et al., 2023).

The need for targeted growth factor delivery to tissues to prevent off target and other adverse systemic effects was addressed by the article of Fok et al. The authors attempted to improve bone morphogenetic protein-2 (BMP-2) retention and presentation by cells using a technique called micromolecular crowding (MMC), which mimics the structure of native cellular microenvironment, using an MMC agent, λ -carragreenan, a red seaweed-derived polysaccharide, in combination with detergent-free decellularization. Using this approach, they were able to significantly improve mineralized tissue formation in a murine model suggesting that this platform could be used to improve the effectiveness of BMP-2 and other growth factor-based therapies.

A strategy for improving osteogenic activity of human mesenchymal stromal cells (hMSCs) was presented by the article of Kolliopoulos et al. The results show that collagen scaffolds incorporating placental products—natural matrices derived from amnion and chorion placental membranes—into the scaffold’s architecture, induce powerful osteogenic response and enhance immunomodulatory and angiogenic potential of hMSC *in vitro*.

On the topic of wound healing, the study of Wu et al. addressed the need for low-cost, biocompatible skin dressings that can adapt to complex mechanical demands of severe skin wounds and promote healing. To this end, the authors generated a hybrid membrane composed of hot-pressed flat silk cocoons (FSC) crosslinked with carboxymethyl chitosan (CMCS) that mimic the 3-dimensional structural components of the skin. The FSC/CMCS substrates exhibit excellent biocompatibility and mechanical properties. Furthermore, they reduce inflammatory cell infiltration, and promote neovascularization in full-thickness skin wounds in a rat model.

Lu et al. reviewed the state of the art and future promise of native and engineered extracellular vesicles (EVs) as therapeutic agents for tissue regeneration and wound healing. They describe how native nano sized EVs derived from different cell types, such as blood cells, skin, and endothelial cells, can be used to deliver a cargo of various growth factors and immunomodulatory molecules to injured cell populations and tissues. They point out that advantages of native EVs as therapeutic agents lie in their excellent biocompatibility as well as in their low immunogenicity and toxicity. Among native EVs’ disadvantages are their low concentration in tissues, a low payload capacity, and a short half-life *in vivo*. To address these issues, engineered EVs have been explored as alternatives to native EVs. The engineering approaches include genetic EV modifications, direct EV membrane modification and combining EVs with nanomaterials.

Another review article in the series by Kozan et al. surveyed design criteria for biomaterial scaffolds to promote endogenous skeletal muscle regeneration to aid in recovery from traumatic muscle injury. Although skeletal muscle possesses the inherent ability to regenerate small-scale injuries, larger injuries (volumetric muscle injuries) heal poorly and result in a severe loss of muscle functionality. The article focuses on the use of biomaterial scaffolds that mimic endogenous skeletal muscle tissue microenvironment and can be implanted into the site of injury to promote regeneration of muscle tissue. The authors argue that the major criteria for the design of such scaffolds, which can be comprised of natural or synthetic biomaterials, should be their porosity, interconnectivity of the pores, alignment of the material comprising the scaffold, and the biomaterial’s mechanical properties. The optimization of these parameters is essential for achieving a robust cellular response which includes myoblast infiltration, myofiber ingrowth and nutrient transfer during skeletal muscle regeneration.

Cell tracing, tissue biosensing and imaging strategies to aid in wound healing and tissue regeneration are addressed in two review articles in this series. Short et al. discussed novel approaches for non-invasive longitudinal monitoring and imaging of chronic wounds - an area of a strong need, as non-invasive methods are still not commonplace in clinical practice. The discussion includes the survey of the bioengineering advances in non-invasive monitoring of the chronic wounds; imaging and modeling wounds’ ECM, and machine learning algorithms as well as other computational tools for evaluation and integration of different sets of data to allow prediction of healing outcomes and planning therapeutic intervention.

It is well-recognized that the migratory behavior of different cell types, including stem and progenitor cells, immune cells, and endothelial cells, among others, strongly influence tissue regenerative outcomes. Accordingly, Hu et al. focused on the methods for quantification of individual cell migration. The authors discuss the analytical metrics characterizing this process, such as cell trajectory, velocity, and migration angles, and highlight the experimental approaches for a quantitative assessment of these metrics *in vitro* and *in vivo*. Additionally, they consider quantitative models of cell migration, and the application of such models to elucidation of the role of cell migration in the biological function.

The breadth of the themes comprising this article collection illuminates the complexity of the field and the many questions to be addressed for achieving effective control of tissue microenvironment for optimizing wound healing and regeneration. The concentration on musculoskeletal tissues in this assemblage mirrors the current focus of the field on regeneration of structural components. Additionally, our collective understanding of inflammation, the foreign body response to implantation, and the triggers of the diverse mechanisms of host immunity and their impacts on tissue regeneration accentuates the need for multi-disciplinary research in a field with such diverse inputs. Undoubtedly, advanced biomaterials and bioengineering tools will play a key role in reaching these goals (Hao et al., 2022; Monaghan et al., 2023). We are only at the beginning of this exciting journey.

## References

[B1] Evano B. Sarde L. Tajbakhsh S. (2023). Temporal static and dynamic imaging of skeletal muscle *in vivo* . Exp. Cell Res. 424, 113484. 10.1016/j.yexcr.2023.113484 36693490

[B2] Freedman B. R. Hwang C. Talbot S. Hibler B. Matoori S. Mooney D. J. (2023). Breakthrough treatments for accelerated wound healing. Sci. Adv. 9, eade7007. 10.1126/sciadv.ade7007 37196080 PMC10191440

[B3] Hao D. Lopez J. M. Chen J. Iavorovschi A. M. Lelivelt N. M. Wang A. (2022). Engineering extracellular microenvironment for tissue regeneration. Bioeng. (Basel) 9, 202. 10.3390/bioengineering9050202 PMC9137730 35621480

[B4] Lumelsky N. (2021). Creating a pro-regenerative tissue microenvironment: local control is the key. Front. Bioeng. Biotechnol. 9, 712685. 10.3389/fbioe.2021.712685 34368106 PMC8334550

[B5] Monaghan M. G. Borah R. Thomsen C. Browne S. (2023). Thou shall not heal: overcoming the non-healing behaviour of diabetic foot ulcers by engineering the inflammatory microenvironment. Adv. Drug Deliv. Rev. 203, 115120. 10.1016/j.addr.2023.115120 37884128

